# Development of a transtheoretical model for the prevention and treatment of skin disease through ultraviolet protection based on 14,681 samples of outpatient clinics and a cross-sectional survey

**DOI:** 10.3389/fpubh.2025.1635806

**Published:** 2025-08-15

**Authors:** Xiaoyi Liu, Guiling Xu, Weili Yang, Danping Su, Renlong Yin, Pengjie Wan, Dan Deng, Ying Xiang, Li Hong

**Affiliations:** ^1^Department of Dermatology, Shanghai Children’s Medical Center, Shanghai Jiao Tong University School of Medicine, Shanghai, China; ^2^Department of Dermatology, Fujian Children’s Hospital (Fujian Branch of Shanghai Children’s Medical Center), College of Clinical Medicine for Obstetrics and Gynecology and Pediatrics, Fujian Medical University, Fuzhou, China; ^3^Nursing Department, Shanghai Children’s Medical Center, Shanghai Jiao Tong University School of Medicine, Shanghai, China; ^4^Department of Dermatology, People’s Hospital of Ning’er Hani and Yi Autonomous County, Pu’er City, China; ^5^Department of Clinical Nutrition, Shanghai Children’s Medical Center, Shanghai Jiao Tong University School of Medicine, Shanghai, China; ^6^Outpatient Department, People’s Hospital of Ning’er Hani and Yi Autonomous County, Pu’er City, China; ^7^Department of Laboratory Medicine, Shanghai Children’s Medical Center, Shanghai Jiao Tong University School of Medicine, Shanghai, China

**Keywords:** skin disease, UV radiation, protection literacy, KAP model, photoprotection

## Abstract

**Background:**

Little is known about the association between types of skin and subcutaneous diseases and UV protection literacy.

**Objective:**

To assess the burden and treatment of skin diseases in Ninger County, Yunnan Province, and establish a transtheoretical model for optimizing public perceptions and behaviors regarding UV radiation.

**Methods:**

Electronic medical records of outpatients from the dermatology department were collected from January 2022 to July 2024, and a self-administered questionnaire assessed the public knowledge about UV exposure risks and behaviors. Multivariable regression models were used to investigate the correlation between UV protection literacy and dermatological situations. Knowledge, Attitude, and Practice (KAP) model was established and re-examined by Structural Equation Modeling (SEM).

**Results:**

Significant seasonal trends in skin disease incidence were observed. Each unit increase in UV protection knowledge score was associated with a 59.1% lower risk of pigmented skin disease and a 56.1% lower risk of skin tumors. A KAP model was developed with coefficients of 0.264 (*p* < 0.001), 0.603 (*p* = 0.041), 0.605 (*p* < 0.001). The impact of redundant residuals on the evaluation of the model fit cannot be ignored in SEM.

**Conclusion:**

The negative association between UV protection literacy and certain skin disease occurence revealed the essentiality of UV protection dissemination.

## Introduction

A critical area of concern is the impact of ultraviolet (UV) radiation on skin health. Intense exposure to medium-wave ultraviolet (UVB) can lead to epidermal necrosis and pigmentation, while UVA can penetrate the epidermis and act on the superficial dermis, which is related to skin aging (1, 2). UV radiation has been shown to contribute to a range of skin disease, which is not only the main spectrum that causes photodermatosis but also a cause of other skin disease such as summer dermatitis, melasma, freckles, lupus erythematosus, actinic keratosis, and squamous cell carcinoma (3–5).

In recent years, the incidence of various skin diseases in primary outpatient care settings has garnered increasing attention from public health officials and researchers. Studies showed that in 2019 there were approximately 485 million new cases of skin and subcutaneous diseases worldwide, with fungal and bacterial skin disease accounting for the majority, at 34 and 23%, respectively. From 1990 to 2019, there was a significant increase in the global number of new cases and deaths from all types of skin and subcutaneous diseases, as did in the burden measured by disability-adjusted life years (DALYs), especially in countries with a low to medium Sociodemographic Index (SDI) level (6). Effective monitoring of skin disease trends is crucial for understanding the evolving landscape of public health issues, particularly in high-risk regions like Yunnan Province, where specific challenges such as geographical diversity and cultural practices pose unique risks (7).

Yunnan Province, located in southwest of China, situated in a mid-low latitude plateau region, experiences weaker absorption and scattering of ultraviolet radiation due to its high altitude and thinner atmosphere in some areas, allowing more ultraviolet radiation to reach the ground. Additionally, as Yunnan is in the southwest monsoon area, the monsoon activity brings warm and moist air streams that make the atmosphere unstable, leading to strong convection and cloudy weather, which suppresses the formation of ground-level ozone. This also results in a weakened ability of the ozone layer to absorb ultraviolet radiation, causing more ultraviolet rays to reach the Earth’s surface. The characteristics of ultraviolet radiation in Yunnan are influenced by various factors, including geographical location, altitude, climate features, atmospheric environment, and topographical structure, making the ultraviolet radiation intensity in Yunnan higher compared to other regions in China. It has an average annual sunshine duration of about 2025.7 h and an average annual ultraviolet index of 10.2 in 2023, indicating that the region enjoys long hours of sunlight and strong ultraviolet radiation (8).

Despite the known risks associated with UV exposure, there remains a significant gap in public knowledge and protective behaviors related to UV radiation. Previous studies have highlighted this discrepancy, emphasizing the urgent need for increased public awareness and education regarding UV protection. To address these issues, the Knowledge, Attitude, and Practice (KAP) model provides a valuable theoretical framework to be examined (9). This model posits that an individual’s inherent knowledge about a health issue influences their attitudes toward that issue, which in turn affects their behaviors. Understanding this triad is vital for developing effective interventions aimed at reducing the incidence of skin disease linked to UV exposure.

Thus, we aimed to (1) analyze the seasonal patterns of skin disease incidence and main treatments in primary outpatient care settings in Yunnan Province over a 2.5-year period, aiming to identify trends and correlations that could inform public health strategies and resource allocation to prevent skin disease. (2) To understand the status quo of questionnaire survey of UV protection, further in the subset of samples to investigate the relationship between knowledge and behaviors related to UV radiation protection and the occurence of specific skin disease. (3) To innovatively develop a preliminary cross-theoretical model of knowledge, attitude, and practice regarding UV protection in order to enhance vulnerable community understanding and practices related to UV protection, ultimately reducing the incidence of skin disease linked to UV exposure.

## Methods

### Outpatient data collection

Electronic medical records of outpatients from the dermatology department of a secondary hospital in Ning’er County, Yunnan Province, over approximately 2.5 years (from January 1, 2022, to July 1, 2024, totaling 912 days) were collected, including demographic information (age, gender, occupation and geographic location), consultation times, diagnostic results, treatment plans, and so on.

### Questionnaire survey

We developed a self-administered questionnaire (see in Supplementary Table S1) to assess knowledge about skin disease, UV exposure risks, and protective behaviors among the randomly selected patients and their family members who visited the dermatology outpatient clinic of a secondary hospital in Ning’er County, Yunnan Province, the southwest of China, from March 2024 to June 2024. After internal consistency testing, the Cronbach’s α coefficient of the overall scale questionnaire is 0.622. The on site form of distributing and collecting the questionnaires was adopted, and the content of the survey was explained to them and informed consent was obtained before the survey. The questionnaire consisted of 5 general information questions and 14 questions on UV protection knowledge and behaviors: including 8 subjective questions and 6 objective ones. Scoring for the objective questions is as follows: 1 point for a correct answer, and no points for a wrong answer. A score of 4 or more is considered passing.

### KAP model development

Utilize findings from the surveys to recreate a KAP model specific to UV protection and skin disease prevention. The model included questions designed to assess participants’ knowledge of UV radiation, attitudes and current practices regarding UV protection. Knowledge referred to the public’s comprehension of the UV damage and correspondent protection and was measured by 5 items. The items were responded with 2-point scale: 1 referred to “Known,” 0 referred to “Partly known/Unknown.” Higher total correct answer to the five knowledge items indicated a higher knowledge level. Attitude referred to the public’s preconception of sun protection at the starting stage and the season required for sun protection. The items were responded with 2-point scale: 1 referred to “Agree,” 0 referred to “Disagree.” Higher scores indicated a more correct and higher attitude level. Multiple practices were measured by 7 items. Participants were asked about the average daily duration of their sun exposure, the main time period they are exposed to the sun daily, the number of times they had sunburns in the past year, the main places where they experienced sunburns, their primary methods of treating sunburns, the sun protection measures they usually take, and the channels through which they obtain knowledge about sun protection.

### Data analyses

Referring to the 9th edition of Dermatology and Venereology, the disease types were reclassified into 21 categories, and descriptive statistical analysis was carried out to understand the basic characteristics of patients and the distribution of diseases. Use time-series analysis to identify seasonal trends in disease incidence.

Descriptive analysis of the overall and individual question correct response rates for the objective questions was conducted. Inter-group comparisons were made using chi-square tests or analysis of variance (ANOVA) or the Kruskal-Walli’s test (non-parametric equivalent of the ANOVA). And multiple linear regression analysis was employed to explore the factors influencing the level of UV protection knowledge score. Further, patient outpatient records were matched, and multivariable linear and logistic regression models were used to investigate the correlation between patients’ UV protection knowledge and behaviors and their dermatological consultation situations, with the adjustment of age and gender. Missing data was handled with single-value random imputation.

Multiple linear regression analysis with the stepwise method was adopted to explore the overall and mediating effects among the KAP model of UV Protection Literacy. We utilized Maximum Likelihood estimation method to determine the parameters in Structural Equation Modeling (SEM), grounded in a theoretical foundation that encompasses path analysis, factor analysis, and regression analysis. Matrices involved the loading matrix, structural coefficient matrix, factor loading matrix, and possible error terms. Additionally, to examine the model fit, the model was identified by ascertaining the path directions, standardized estimates, Bentler-Bonett normed fit index (NFI), relative fit index (RFI), incremental fit index (IFI), comparative fit index (CFI), and root mean square error of approximation (RMSEA). The model was judged to fit the data well if NFI, RFI, IFI, and CFI are greater than 0.90 and RMSEA was less than 0.10. The required significance level (alpha) for all tests was 0.05.

### Quality control

We verified the data entry process and conducted checks for data consistency and completeness from the electronic medical records. The UV protection literacy survey was conducted using a non-biased, convenient sampling method to gather data, being partially representative of the population of interest. The internal consistency of the scales and the intended constructs were checked to capture the reliability of the measures. We also verified that statistical assumptions like normality, homoscedasticity, and independence of errors underlying the mediation analysis were met and evaluated the fit of the SEM model to the data involving various fit indices.

## Results

### Outpatient visit patterns in dermatology at primary hospitals in Yunnan

**Figure 1:**
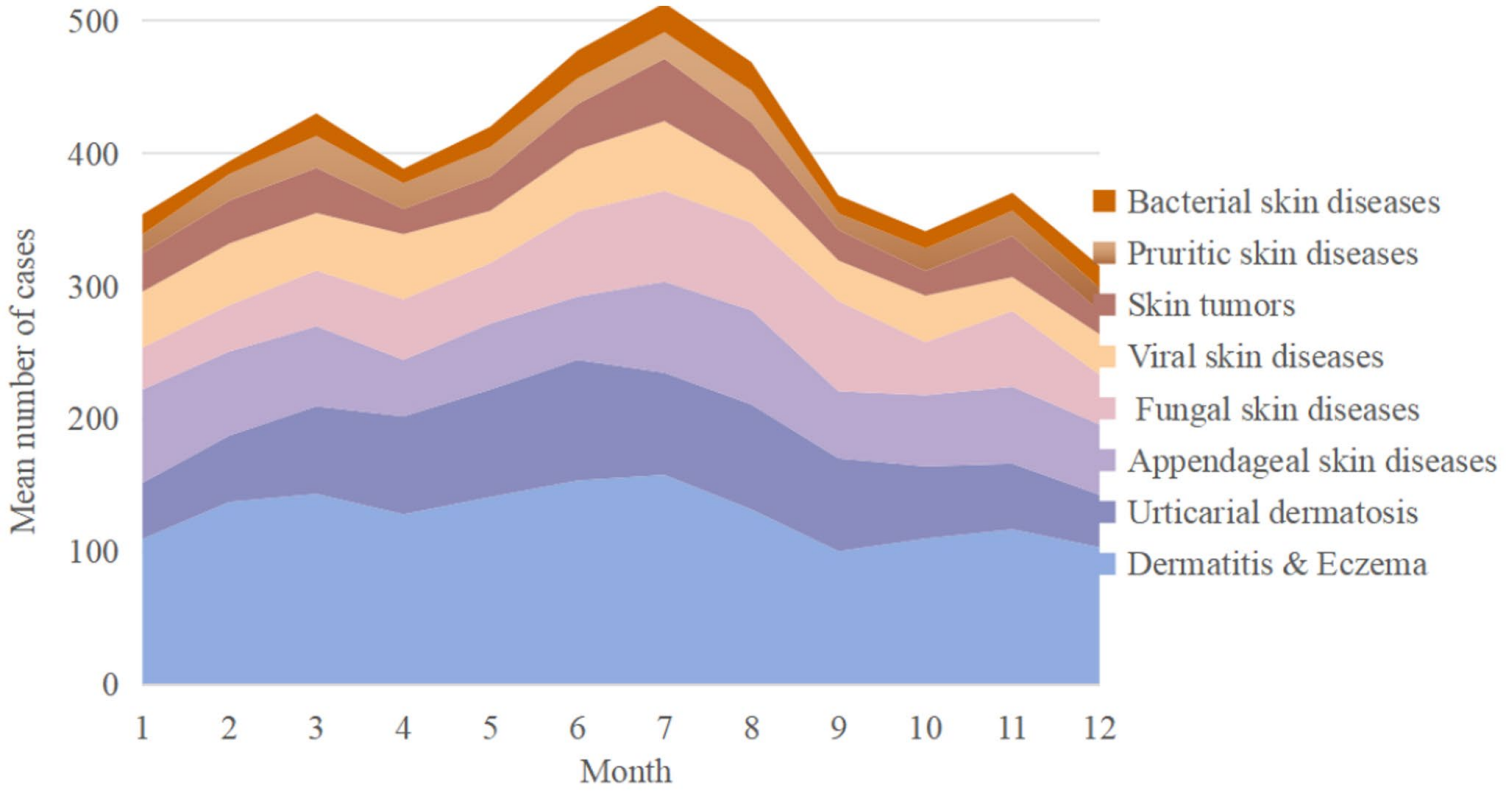
Mean cases of various skin disease types per month.

The average number of outpatient visits per month was 489 cases, and the mean daily average was 16 cases. The average patient age was 34.1 years, with 53.4% being female and 56.2% identifying as Han ethnicity. A majority, 81.6%, were first-time visitors, while 21.1% had a recorded past medical history (Supplementary Table S2). Table 1 demonstrates the top five skin diseases in outpatient clinics and their main treatment measures. The stacked area chart (Figure 1) illustrates the cyclical fluctuations in the number of cases for the top eight major skin diseases throughout the years.

**Table 1:** Top five skin disease classification and treatment characteristics among outpatients.

Classification	Cases, *N*	Numbers of medical advice	Main treatment measures
Dermatitis and eczema	3,885	2.80	Oral anti-allergic drugs (*n* = 3,295) Drugs for external use (*n* = 3,526) Diet advice (*n* = 928) Sun protection and moisturizing (*n* = 730) Using corticosteroids (*n* = 531)
Urticarial dermatosis	1,951	2.46	Oral anti-allergic drugs (*n* = 1,681) Drugs for external use (*n* = 1,219) Regular follow-up visits(*n* = 846) Avoiding all kinds of stimuli (*n* = 238) Oral administration of traditional Chinese medicine (*n* = 205)
Appendageal skin diseases	1,711	2.72	Drugs for external use (*n* = 1,310) Sun protection and moisturizing (*n* = 924) Diet advice (*n* = 836) Acid peeling (*n* = 256) Oral antibiotics (*n* = 219)
Fungal skin diseases	1,466	2.21	Drugs for external use (*n* = 1,017) Completion of tests and examinations (*n* = 922) Sanitation and disinfection (*n* = 321) Oral antifungal (Dafukang) (*n* = 117) Avoiding smoking and alcohol (*n* = 107)
Viral skin diseases	1,225	2.34	Physical therapy(*n* = 541) Antiviral therapy (*n* = 426) Oral medicine (*n* = 367) Waterproof and moisture-proof (*n* = 330) Regular follow-up visits (*n* = 323)

### UV protection questionnaire survey

A total of 526 questionnaires were recovered. Socio-demographic characteristics of participants enrolled in UV protection survey questionnaire can be seen in Supplementary Table S3.

The average score was 2.35 with a SD of 1.66. A total of 145 people achieved a passing score, accounting for 27.6% of the total, and 13 people scored full marks, which is 2.5% of the total. Table 2 presents the correct response rates for each item of the objective questions. Supplementary Table S4 displays the comparison of UV protection knowledge scores among participants with different demographic characteristics, showing significant differences in gender, age, and occupation (*p* < 0.001), while there was no significant difference in place of residence (*p* = 0.078). Supplementary Table S5 exhibits performance on, respectively, objective items. The results from multiple linear regression analysis show that gender, age, occupation, and place of residence all contributed to the level of correct understanding of ultraviolet protection knowledge among the surveyed individuals. Male and older age were associated with a lower level of correct understanding of ultraviolet protection knowledge (Supplementary Table S6).

**Table 2:** Significant association between UV protection knowledge score and skin health outcomes from multiple regression analysis*.

Dependent variable	Independent variable	β	S. E.	*P*	EXP(β)	95%CI	
						Lower limit	Upper limit
Pigmented skin disease	UV protection knowledge score	−0.894	0.437	**0.041**	0.409	0.174	0.964
Skin tumors	UV protection knowledge score	−0.823	0.416	**0.048**	0.439	0.194	0.992

*Adjusted for age and sex.

*P* values < 0.05 are shown in bold.

As shown in Supplementary Table S7, there were significant differences (*p* < 0.05) in sun exposure duration, the number of sunburns and their treatment methods in the past year, and the level of understanding of sun protection knowledge. As seen in Supplementary Table S8, the main daily sun exposure period for the surveyed individuals is from 10 a.m. to 4 p.m. The primary locations for sunburn occur during outdoor activities. The top three usual sun protection measures are wearing sun hats/using umbrellas, wearing long-sleeved shirts and pants, and using sunscreen. The sources for obtaining sun protection knowledge are ranked as follows: the Internet, television/newspapers/publications, doctors, colleagues (classmates)/parents, and schools.

After adjusting for age and sex in the logistic regression model, an increase in the number of skin disease visits was associated with an increase in the number of sunburns occurred in the past 1 year (β = 0.207, *t* = 4.845, *p* = 0.041, not shown in tables). There was also a significant negative correlation between the UV protection knowledge score and the occurrence of two types of skin disease (Table 2). The risk of pigmented skin disease (e.g., vitiligo) was reduced by 59.1% and the risk of skin tumors (e.g., keloids) was reduced by 56.1% for each increased unit of UV protection knowledge score.

### Analysis of mediating effects and transtheoretical model

The independent variable “Knowledge” has a significant impact on the dependent variable “Practice” (β = 0.207, *p* < 0.001), indicating that the total effect is established (Model 1). “Knowledge” has a significant impact on the mediator variable “Attitude” (β = 0.264, *p* < 0.001) in Model 2. Meanwhile, in Model 3 “Knowledge” has a significant impact on “Practice” (β = 0.605, *p* < 0.001), and “Attitude” has a significant impact on “Practice” (β = 0.603, *p* = 0.041) (Table 3; Supplementary Figure S1).

**Table 3:** Mediating effects among knowledge, attitude, and practice model.

Model	Model 1			Model 2			Model 3		
Variable	Practice			Attitude			Practice		
	β	*t*	*P*	β	*t*	*P*	β	*t*	*P*
Knowledge	0.207	4.845	**<0.001**	0.264	11.332	**<0.001**	0.605	3.446	**<0.001**
Attitude							0.603	2.049	**0.041**
*R* ^2^	0.043			0.197			0.05		
Adjusted *R*^2^	0.041			0.195			0.047		
*F*	23.469			128.411			13.904		
*P*	**<0.001**			**<0.001**			**<0.001**		

*P* values < 0.05 are shown in bold.

We further conducted structural equation modeling based on the theoretical foundation which considered the potential residuals in the models (Supplementary Figure S2, variables and results assignment in the KAP model were in Supplementary Table S9). However, in Table 4, results were partially consistent with the mediation model, with a non-optimal model fit of NFI 0.797, RFI 0.750, IFI 0.832, CFI 0.830, RMSEA 0.085 [90% confidence interval: 0.073–0.097] after 49 model iterations in procedure.

**Table 4:** Path coefficients in the knowledge-attitude-practice framework in SEM.

Variable	Path	Variable	Estimate	S. E.	C. R.	*P*	Label
Attitude	←	Knowledge	0.553	0.102	5.413	**<0.001**	B
Practice	←	Attitude	0.355	1.050	0.339	0.735	C
Practice	←	Knowledge	−0.178	0.591	−0.301	0.763	A

*P* values < 0.05 are shown in bold.

## Discussion

In this study, we firstly assessed the burden pattern of skin disease in terms of time and main treatment measures in Ninger Hani and Yi Autonomous County over 900 days among 14,681 outpatients and applied a self-designed questionnaire survey to investigate the public perceptions and behaviors regarding the UV radiation, and further correlated the survey findings and dermatological visits. We innovatively constructed a transtheoretical KAP model for the prevention and treatment of skin disease through ultraviolet protection, providing a new perspective for understanding the inner relationship between public awareness of UV damage and behaviors and reverse the progression of disease.

The outpatient data revealed significant seasonal trends in skin disease incidence, with particular peaks noted during specific months and relatively stable during other months. Summer is the peak period for the number of skin disease cases, which may be related to high temperatures, humidity, increased outdoor activities, and longer exposure to sunlight, consistent with the previous discovery (10). It is worth noting that, despite the rainy season in Ninger from June to August, the high level of scattered radiation, rich in UVA and UVB, is ubiquitous in the environment (11). The increase in bacterial skin disease and pruritic skin disease during summer may be associated with increased sweating and skin moisture, providing a favorable environment for the growth of bacteria and parasites (12, 13).

One important aspect of certain disease incidence is the role of seasonal patterns, which are intricately linked to climate and environmental factors. As such, understanding these seasonal fluctuations is essential for crafting effective public health interventions that are responsive to the needs of the community throughout the year. The results can inform the collaboration with local health authorities to discuss medical resource allocation based on identified trends, such as adding the special outpatient service and convenient treatment channels. These findings partly aligned with existing literature that suggests seasonal variations in disease incidence are influenced by climatic factors, as well as socio-economic conditions such as population density and healthcare access (14). The Singapore study in elevated concentrations of PM_2.5_ and PM_10_, along with increased rainfall, are significantly positively associated with the risk of outpatient visits for atopic dermatitis (15). However, in our study, certain diseases, such as skin tumors, did not show clear seasonal patterns, indicating potential gaps or variations in local environmental conditions and data collection that warrant further investigation. This highlights the need for more localized and representative studies to understand the unique epidemiological landscape of Yunnan and the factors influencing these trends in the term of skin diseases.

It is also noticeable that distinct therapeutic approaches were conducted on the basis of different types of skin conditions according to the local medical resources and economic status. For dermatitis, eczema and urticarial dermatosis, the most common treatments are oral anti-allergic drugs and topical drugs (e.g., Mometasone Furoate Cream, calamine Lotion). In dermatitis and eczema, the education of emphasizing sun protection and moisturizing is also prominent, indicating the skin care awareness of local dermatologists. It is hard to ignore that in dermatitis and eczema diseases, the systematic use of glucocorticoids ranks the fifth treatment measure. Glucocorticoid is a low-cost and fast-acting anti-inflammatory drug. And local patients’ fear of glucocorticoids is not as strong as that of patients in developed regions. We think that it is related to local economic conditions, patients’ desire for rehabilitation and the less attention to side effects of glucocorticoids. In urticarial skin diseases, oral Chinese medicine treatment is also in the forefront. Biomedicine and public health industry have been the major industries in Yunnan since 2016, and more than 2,000 ethnic medicinal resources and more than 10,000 folk prescriptions are native to Yunnan (16). In the diagnosis and treatment of fungal skin diseases, fungal fluorescent detection has been carried out which improving the positive detection rate. Due to the limitations in medical resources, fluconazole is usually used as an oral antifungal drug. Moreover, as local drinking habits are common, dermatologists will not only prescribe oral antifungal drugs but also advise patients not to smoke or drink. Viral skin diseases are mainly warts, herpes simplex and herpes zoster, and the treatment measures are similar to other areas in China.

Our survey revealed that while a majority of respondents were aware of the harmful effects of UV exposure, only a minority consistently practiced effective protective behaviors, such as using sunscreen or wearing protective clothing. Demographic analysis indicated that factors such as age, gender, and education level significantly influenced knowledge and behavior patterns, consistently established by previous studies (17, 18). The results of the survey also underlined the implications of the gap between knowledge and practice on skin disease prevalence. The significant inverse correlation between the UV protection knowledge score and the incidence of two types of skin diseases (pigmented skin disease and skin tumors) in our study highlighted the importance of residents’ UV protection knowledge and literacy for skin health. Despite awareness of UV risks, behavioral discrepancies may stem from socio-economic barriers, such as the cost of sunscreen and protective clothing (19), or cultural beliefs that minimize the perceived importance of UV protection (20, 21). Addressing these barriers through targeted interventions could significantly enhance public health outcomes. The systematic review by Collins LG et al. concluded from the included 12 studies with reporting quality that highly favorable returns the investment and substantial reduction on skin cancers were found from a societal cost perspective of multi-component sun safety campaigns and other relevant interventions (22). Hence, this was considered to be valid that in low and middle-income countries, the involvement of local stakeholders and the role of tailored processes were adapted to promote low-cost sun protection measures in order to enhance the public awareness of UV protection (23).

In response to the identified gaps, we further developed a trans-theoretical model to re-integrate knowledge, belief, and behavior regarding UV protection and skin health. This model provided a inner-restructured approach to understand behavior change, categorizing individuals based on their readiness to adopt various protective measures to be free from UV radiation damage (24). Proposed intervention strategies based on this model include community education programs, workshops aimed at increasing awareness, and social media campaigns to disseminate information on UV protection. These interventions will target identified knowledge and behavior gaps, with anticipated impacts including increased knowledge retention, improved understanding of UV risks, and enhanced adoption of protective behaviors. As a basic framework that helps understand the interplay between individuals’ knowledge, their attitudes, and the resultant behaviors, the model is particularly useful in fields such as health education, marketing, and social sciences, and still need to be adjusted by culture and region difference within the primary care setting (25, 26). Furthermore, exploring a cross-theoretical approach can enhance the effectiveness of future interventions by integrating insights from various health behavior theories, such as ABCDE theory (Activating Event, Beliefs, Consequences, Disputing, Effect) (27). Moreover, despite the suboptimal model fit of SEM model, our core findings retain theoretical significance. We find that the 90% confidence interval for RMSEA [0.073–0.097] does not exceed the 0.10 threshold, indicating the basic structural rationality of the model and supporting its fundamental usability.

Despite the advantages in collecting extensive dermatology data and innovatively constructing the KAP model, limitations of the study include potential regional normative differences in skin disease treatment and the need for questionnaire validation. This study has not fully considered the normative differences of skin disease in primary medical treatment in different regions, which may affect the consistency and comparability of the data. Moreover, while the construction of the structural equation model itself is part of the confirmatory factor analysis in construct validity, the test–retest reliability and the stability of results across different cultural and demographic groups were not simultaneously examined and may influence the interpretation of extrapolation and extrapolation application of the results. Moving forward, we plan to build on this correlational analysis by designing a prospective cohort study or intervention study, allowing us to strengthen causal inferences regarding how knowledge acquisition drives behavioral changes over time, ultimately refining targeted health literacy interventions.

In summary, the present study analyzed seasonal patterns of skin disease incidence and corresponding treatment measures in the primary medical areas of Yunnan Province, investigated the relationship between knowledge, behaviors, and skin disease prevalence, and developed a KAP model for UV protection. Our finding found that the significant association between the UV protection literacy and the skin health, further underscore the critical need for effective interventions to enhance the consciousness of UV radiation protection and prevent skin disease.

## Data Availability

The raw data supporting the conclusions of this article will be made available by the authors, without undue reservation.
