# Supplementary material for: Development of a transtheoretical model for the prevention and treatment of skin disease through ultraviolet protection based on 14,681 samples of outpatient clinics and a cross-sectional survey
Source: Front Public Health. 2025 Aug 15;13:1635806. doi: 10.3389/fpubh.2025.1635806 (PMC12394189; doi:10.3389/fpubh.2025.1635806)
Supplement: Supplementary file 1 [file Table_1.docx]

**Supplement Tables**

Table S1: Survey Questionnaire on Sun Exposure Hazards and Protection Measures

| No. | Title | Response |
| --- | --- | --- |
| 1 | Gender | □ Male □ Female |
| 2 | Age | ______ years |
| 3 | Mobile phone number | ___________ |
| 4 | Your occupation | □ Office worker □ Laborer □ Farmer □ Self-employed □ Retired □ Student / Non-residential Child |
| 5 | Place of residence | □ Ning'er County □ Simao District □ Jinggu County □ Jinghong City  □ Other (Please specify: ______) |
| 6 | What are the main hazards of UV radiation to the human body? (Multiple choice) | □ Skin tanning □ Skin aging □ Skin cancer □ Eye damage |
| 7 | Which type(s) of UV radiation cause(s) skin damage? | □ UVA □ UVB □ UVA+UVB □ Don't know |
| 8 | At which life stage do you believe sun protection should begin? | □ Not necessary □ Childhood □ Adolescence□ Adulthood □ Middle/Old age |
| 9 | In which seasons is sun protection necessary in your area of residence? (Multiple choice) | □ Spring □ Summer □ Autumn □ Winter □ Not necessary |
| 10 | What is your average daily cumulative sun exposure duration? | □ <1 hour □ 1~2 hours □>2 hours |
| 11 | What are your main daily sun exposure time periods? (Multiple choice) | □ 8:00 AM - 10:00 AM □ 10:00 AM - 4:00 PM □ 4:00 PM - 6:00 PM |
| 12 | How many times did you experience sunburn in the past year? | □ 0 times □ 1 time □ 2 times or more |
| 13 | Where did your sunburns primarily occur? (Multiple choice) | □ Outdoor activities □ Swimming pool or beach □ Other (Please specify: ______) |
| 14 | How do you primarily treat sunburn? | □ No treatment □ Self-apply topical cream/bought from pharmacy □Seek help from friends/parents □ Hospital visit □ Private clinic □ Online consultation |
| 15 | What sun protection measures do you routinely use? (Multiple choice) | □ Use sunscreen □ Wear sun hat/Use umbrella □ Wear sun-protective mask □ Wear long sleeves/pants □ Stay in shade □ Wear sunglasses |
| 16 | Do you understand the labeling on sunscreen products? | □ Understand □ Partially understand □ Do not understand |
| 17 | How long before going outdoors do you typically apply sunscreen? | □ Immediately before going out □ 15-30 minutes before going out □ Never apply □ Reapply every 3-4 hours □ Reapply after sweating |
| 18 | . Which special environments require UV protection? (Multiple choice) | □ Cloudy days □ Indoors  □ Snowy areas □ Seaside |
| 19 | Through which channels do you acquire knowledge about sun protection? (Multiple choice) | □ Internet □ TV/Newspapers/Magazines □ Doctor □ School □ Colleagues/Classmates/Parents |

Table S2: Demographics of Patients（N=14681）

| Variable | n | % |
| --- | --- | --- |
| Age，yrs | 34.1 | 21.3 |
| Gender  Male  Female | 6841  7840 | 46.6  53.4 |
| Ethnicity  Han  Others | 8247  6434 | 56.2  43.8 |
| Visit Type  First Visit  Follow-up Visit | 11973  2708 | 81.6  18.4 |
| Past Medical History  Yes  No | 3092  11589 | 21.1  78.9 |
| Month of Visit  Jan.-Mar.  Apr.-Jun.  Jul.-Sep.  Oct.-Dec. | 4263  4601  3241  2576 | 29.0  31.3  22.1  17.6 |

Table S2: Socio-demographic characteristics of the participants (N=526)

| Variable | N | % | $\overline{X}$ | SD |
| --- | --- | --- | --- | --- |
| Gender  Male  Female | 146  380 | 27.8  72.2 |  |  |
| Age，yrs |  |  | 35.9 | 11.9 |
| Occupation  Clerk  Laborer  Farmer  Freelancer  Retired  Student/Homestay Child  Specialist  Healthcare Worker  Civil Servant | 44  18  96  80  13  46  57  164  8 | 8.4  3.4  18.3  15.2  2.5  8.7  10.8  31.2  1.5 |  |  |
| Place of Residence  Ninger District  Simao District  Jinggu District  Jinghong City  Other district/City | 473  24  8  2  19 | 89.9  4.6  1.5  0.4  3.6 |  |  |

Table S4: Comparison of ultraviolet protection knowledge scores among participants with different demographic characteristics

| Variable | Group | Score on Knowledge of Ultraviolet Protection | F | *P value* |
| --- | --- | --- | --- | --- |
| Gender | Male  Female | 1.73（1.41）  2.59（1.69） | 29.863 | **<0.001** |
| Age | <35  35-55  ＞55 | 2.56（1.63）  2.27（1.68）  1.19（1.201 | 8.810 | **<0.001** |
| Occupation | Clerk  Laborer  Farmer  Freelancer  Retired  Student/Homestay Child  Specialist  Healthcare Worker  Civil Servant | 2.61（1.67）  1.78（1.26）  1.04（1.11）  1.61（1.23）  0.69（0.63）  1.57（1.53）  3.30（1.50）  3.47（1.29）  3.00（1.20） | 38.710 | **<0.001** |
| Place of Residence | Ninger District  Simao District  Jinggu District  Jinghong City  Other district/City | 2.30（1.64）  2.96（1.73）  2.38（1.77）  1.00（1.41）  3.05（1.84） | 2.115 | 0.078 |

Table S5: Performance on Objective Questions

| Item | Number of Correct Answers, n | Correct Rate, % |
| --- | --- | --- |
| Main hazards of ultraviolet rays | 228 | 43.4 |
| Ultraviolet rays that damage the skin | 197 | 37.5 |
| Stage of initiating sun protection | 305 | 58.0 |
| Seasons requiring sun protection | 266 | 50.6 |
| Habit of applying sunscreen before going out | 171 | 32.5 |
| Special environments requiring UV protection | 71 | 13.5 |

Table S6: Impact of Demographic Characteristics on the Score of Ultraviolet Protection Knowledge from Multiple Regression Analysis

| Variables | β | S.E. | t | *P value* |
| --- | --- | --- | --- | --- |
| Constant | -0.266 | 0.349 | -0.760 | 0.447 |
| Gender | 0.825 | 1.140 | 5.876 | **<0.001** |
| Age | -0.311 | 0.108 | -2.880 | **0.004** |
| Occupation | 0.279 | 0.026 | 10.767 | **<0.001** |
| Place of residents | 0.157 | 0.078 | 2.025 | **0.043** |

Table S7: Frequency Differences in Ultraviolet Protection Behavior (Single Choice Question)

| Item | Group | N | % | $\chi^{2}$ | *P* |
| --- | --- | --- | --- | --- | --- |
| Cumulative daily sun exposure duration | ＜1 hour  1～2 hour(s)  ＞2 hours | 152  201  173 | 28.9  38.2  32.9 | 6.894 | **0.032** |
| Number of sunburns in the past 1 year | 0 time  1 time  ≥2 times | 301  78  147 | 57.2  14.8  27.9 | 148.681 | **<0.001** |
| If you have sunburn in the past 1 year, the main treatment is? | Not processed  Rub/buy topical cream  Ask a friend/parent for help  Hospital visits  Private clinics  Online consultation | 57  120  5  47  2  3 | 24.4  51.3  2.1  20.1  0.9  1.3 | 276.154 | **<0.001** |
| Do you know the sunscreen label? | understand  Partially understood  I don't understand | 96 265  165 | 18.3  50.4  31.4 | 82.361 | **<0.001** |

Table S8: Response to Ultraviolet Protection Behaviors (Multiple Choice Questions)

| Item | Option | N |
| --- | --- | --- |
| Primary sun exposure period daily | 8 a.m.～10 a.m.  10 a.m.～4 p.m.  4 p.m.～6 p.m. | 170  381  190 |
| Main places of sunburn occurrence | Outdoor activities  Swimming pools or beaches  Countryside fields  Others | 167  61  116  44 |
| Usual sun protection measures | Use of sunscreen  Wearing sun hats/using parasols  Wearing sun-protective masks  Wearing long-sleeved shirts and pants  Staying in the shade  Wearing sunglasses  None | 277  414  243  297  270  145  28 |
| Channels for acquiring sun protection knowledge | Internet  Television/newspapers/publications  Doctors  Schools  Colleagues (classmates)/parents | 391  270  239  94  193 |

Table S9: Variables and results assignment in the KAP model of UV protection literacy

| Variable | Result assignment | |
| --- | --- | --- |
| **Kn: Knowledge** | | |
| K1: Do you know that the main hazards of ultraviolet light to humans are skin tanning, skin aging, skin cancer and eye damage？ | 1=Known | 0=Partly known/Unknown |
| K2: Do you know that the ultraviolet skin damage light is UVA plus UVB? | 1=Known | 0=Partly known/Unknown |
| K3：Do you know the sunscreen label? | 1=Understood | 0=Partly understood/Misunderstood |
| K4: Whether you know the special environments of the cloudy day, indoor, snow, and the sea need ultraviolet protection? | 1=Known | 0=Partly known/Unknown |
| K5: Do you know that you should apply sunscreen 15-30 minutes before going out? | 1=Known | 0=Partly known/Unknown |
| **At: Attitude** |  |  |
| At1: Do you think suns protection should start from children period? | 1=Agree | 0=Disagree |
| At2:Do you think you need sun protection in the all 4 seasons? | 1=Agree | 0=Disagree |
| **Pr: Practice** | | |
| Pr1: Daily Sun Exposure Duration | 1=＜1 hour；2=1～2 hour(s)；3=＞2 hours | |
| Pr2: Primary Sun Exposure Time Period | 1=8 a.m.～10 a.m.; 2=10 a.m.～4 p.m.; 3=4 p.m.～6 p.m.；4=Two or more periods | |
| Pr3: Frequency of Sunburns in the Past Year | 1=zero；2=1 time; 3=2 or more times | |
| Pr4: Main Locations of Sunburn Occurrence | 0=None; 1=Outdoor activities；2=Swimming pools or beaches；3=Other locations；4=Two or more locations | |
| Pr5: Main Methods of Sunburn Treatment | 1=No treatment; 2=Self-treatment with skincare cream or over-the-counter purchase; 3=Seeking help from friends or parents; 4=Hospital visit; 5=Private clinic; 6=Online consultation | |
| Pr6: Usual Sun Protection Measures | 0=None; 1=Use of sunscreen; 2=Wearing a sun hat/using an umbrella/a sun mask/sunglasses/wearing long sleeves and pants; 3=Staying in the shade; 4=Two or more sun protection measures | |
| Pr7: Sources of Sun Protection Knowledge | 1=Internet; 2=Television/newspapers/magazines；3=Doctors; 4=School; 5=Colleagues (students)/parents; 6=Two or more channels | |
